# A cuckoo-like parasitic moth leads African weaver ant colonies to their ruin

**DOI:** 10.1038/srep23778

**Published:** 2016-03-29

**Authors:** Alain Dejean, Jérôme Orivel, Frédéric Azémar, Bruno Hérault, Bruno Corbara

**Affiliations:** 1 Université de Toulouse, UPS, INP, Laboratoire Écologie Fonctionnelle et Environnement, 118 route de Narbonne, 31062 Toulouse, France; 2 CNRS, UMR EcoLab, 31062 Toulouse, France; 3 CNRS, UMR Ecologie de Forêts de Guyane (AgroParisTech, CIRAD, CNRS, INRA, Université de Guyane, Université des Antilles), Campus agronomique, BP 316, 97379 Kourou cedex, France; 4 CIRAD, UMR Ecologie de Forêts de Guyane (AgroParisTech, CIRAD, CNRS, INRA, Université de Guyane, Université des Antilles), Campus agronomique, BP 316, 97379 Kourou cedex, France; 5 CNRS, UMR Laboratoire Microorganismes, Génome et Environnement, Université Blaise Pascal, Complexe Scientifique des Cézeaux, 63177 Aubière cedex, France; 6 Université Clermont Auvergne, Université Blaise Pascal (LMGE), 63000 Clermont-Ferrand, France

## Abstract

In myrmecophilous Lepidoptera, mostly lycaenids and riodinids, caterpillars trick ants into transporting them to the ant nest where they feed on the brood or, in the more derived “cuckoo strategy”, trigger regurgitations (trophallaxis) from the ants and obtain trophic eggs. We show for the first time that the caterpillars of a moth (*Eublemma albifascia*; Noctuidae; Acontiinae) also use this strategy to obtain regurgitations and trophic eggs from ants (*Oecophylla longinoda*). Females short-circuit the adoption process by laying eggs directly on the ant nests, and workers carry just-hatched caterpillars inside. Parasitized colonies sheltered 44 to 359 caterpillars, each receiving more trophallaxis and trophic eggs than control queens. The thus-starved queens lose weight, stop laying eggs (which transport the pheromones that induce infertility in the workers) and die. Consequently, the workers lay male-destined eggs before and after the queen’s death, allowing the colony to invest its remaining resources in male production before it vanishes.

Due to their enormous ecological success, ant colonies, especially large ones, are major resources for other invertebrates, including a diversity of myrmecophilous taxa^1^,^2^. Although many myrmecophiles are commensals that feed on ant waste, others are parasites that prey on ant workers or ant brood^1^,^3^,^4^,^5^,^6^,^7^. In the “cuckoo strategy”, thought to be derived relative from ant brood predation^8^, the myrmecophile is able to trigger regurgitation, or trophallaxis, from the host ants by using its antennae and/or its forelegs to imitate a soliciting worker antennating the head of a donor. In some cases, even simple contact with the host ant’s labium can be sufficient^1^,^4^,^9^. Although approximate, these interspecific solicitations are rewarded because, in ants, the antennation of the donor head by the soliciting worker is enough for trophallaxis to occur, so that the adaptive process took place only on the myrmecophile side (i.e., there was no parallel evolution on the ant side^10^).

Most ant species have “closed” colonies where colony mate recognition is based on a mixture of low-volatile cuticular hydrocarbons constituting the “colony odor” which, once learned by the workers, represents a neural template. Discrimining between colony mates and aliens is based on the qualitative and quantitative comparison of the hydrocarbons making up the colony odor (i.e., the template) with those of the encountered individuals, a mismatch usually resulting in aggressiveness^11^. The integration of myrmecophiles into ant colonies is based on the chemical similarities of their cuticular profiles with that of their host^7^,^11^ and/or the use of defensive strategies. Chemical mimicry has been noted in lycaenid caterpillars, and the cuticular hydrocarbon profile of certain of them is sufficiently similar to that of the host ant brood to induce workers to carry them into their nests^12^,^13^. Acoustical signals can also be used; the caterpillars can even imitate those of their host ant queens, thus obtaining a high status in the host ant colony hierarchy^14^,^15^,^16^. The caterpillars of some lycaenids and riodinids have evolved several means of defense from ant attacks such as a very thick cuticle, a retractable head, and specialized exocrine glands that inhibit ant aggressiveness and, in certain subfamilies, attract/alert ants plus secrete nutritious compounds. Most of them are herbivorous while others are predators of ant-attended hemipterans and still others are myrmecophagous and prey on the host ant brood or have evolved a cuckoo-like strategy^4^.

Specialized hemipteran feeders have also been noted in several moth families^3^, and the caterpillars of certain species are able to solicit honeydew from hemipterans^17^. Moreover, in the Cyclotornidae, the first instar caterpillars follow the tracks of *Iridomyrmex* ants to feed on attended leafhoppers; second instars trick these ants into transporting them to the nest where they feed on the ant brood and reward the workers with anal secretions^18^. Other cases of moth caterpillars living as brood predators in ant colonies are known^3^, but the cuckoo-like strategy has never before been noted.

Here we report such a case of a cuckoo-like strategy by the caterpillars of the noctuid moth *Eublemma albifascia* in the nests of the African weaver ant *Oecophylla longinoda*. In the genus *Eublemma*, the caterpillars of most species are known to be specialized coccid predators^3^,^19^,^20^ that can be tolerated by the attending ants^21^,^22^; the ability to prey on sap-sucking hemipterans is considered one of the prerequisites in the evolution toward myrmecophily^23^,^24^. In addition to gathering information on the natural history of *E. albifascia*, we quantified the pressure the caterpillars exert on the host ant colonies and its consequences.

## Methods

### Study sites and focal species

Field studies were conducted from 205 *O. longinoda* colonies in Gabon (*Forêt des Abeilles*; 0°40′S, 11°54′E; five colonies) and in different sites in Cameroon: in the city of Yaoundé (03°52′N, 11°31′E; 39 colonies) and its surroundings including Batchenga (03°51′N, 11°42′E; 21 colonies), Kala (03°50′N, 11°21′E; 46 colonies), Matomb (03°53′N, 11°14′E; 34 colonies), Mbalmayo (03°29′N, 11°30′E; 14 colonies), and Nkolbisson (03°59′N, 11°28′E; 32 colonies); and in areas close to Edea (3°47′N, 10°08′E; 11 colonies), Kribi (2°56′N, 9°54′E; seven colonies), Ebolowa (2°54′N, 11°09′E; five colonies) and Buéa (4°09′N, 9°14′E; four colonies).

*Oecophylla longinoda*, of the subfamily Formicinae, is a “territorially dominant arboreal ant species”^1^,^25^,^26^,^27^,^28^ characterized by large, polydomous (multiple nests) arboreal colonies; the workers construct the nests by manipulating larvae to glue leaves together with the silk they produce. Contrary to most formicine ants, the larvae pupate without enclosing themselves in silk cocoons. While plentiful in tree crop plantations where it is used as a biocontrol agent, in natural conditions this species occurs in relatively young or recently disturbed vegetation, whereas very old tropical rainforest trees rather shelter arboreal, carton-nesting *Crematogaster* spp.^1^,^25^,^26^,^27^,^28^.

As for most other ant species, *Oecophylla* workers cannot mate due to the lack of the appropriate apparatuses, but majors have fully functional ovaries and so can lay trophic eggs that are generally given to the queen. They produce unfertilized, chorioned eggs which can develop into fully functional males when the queen dies^29^. Because the colonies are very populous (more than 500,000 individuals^1^), it is difficult to conduct experiments on them. Swarms occur all throughout the year (AD, pers. obs.) which permitted us to compare the production of winged sexuals and moths.

**Figure 1:**
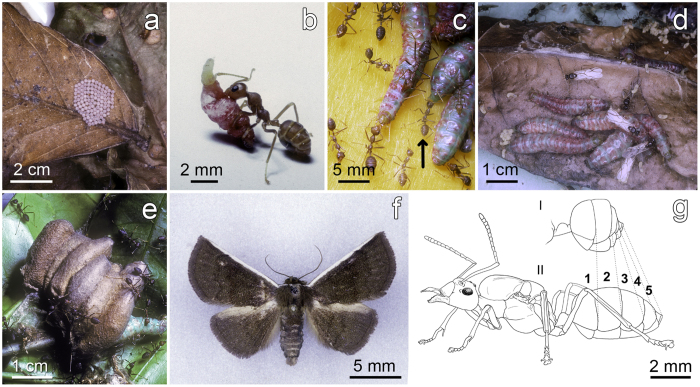
Representation of the different phases in the life cycle of *Eublemma albifascia*. (**a**) Egg cluster (N = 113). (**b**) Second instar caterpillar transported by a minor worker. (**c**) Left, a trophallactic exchange between a last instar caterpillar and a major worker while another worker licks the cuticle of the caterpillar; right (see arrow), a worker imbibes the anal secretion of another last instar caterpillar. (**d**) Last instar caterpillars in a highly parasitized colony with many males. (**e**) A group of agglutinated cocoons. (**f**) An adult moth. (**g**) A drawing representing the contracted gaster of an *Oecophylla longinoda* queen in certain parasitized colony (I) and a “normal queen” in control colonies and other parasitized colonies (II).

In moths, the number of larval instars can vary intraspecifically. In the genus *Eublemma* (Noctuidae; Acontiinae), this number varies from eight to ten^30^,^31^ rendering it difficult to identify the different instars. Therefore, we differentiated only four “steps” in the larval life of *E. albifascia* (see Fig. 1 for the entire life cycle). Newly-hatched caterpillars, or first instars, develop into ≈ 4 mm-long second instars frequently found among the ant brood. The “intermediary” instars are caterpillars 8–20 mm-long able to solicit trophallaxis from workers, the same being true for last instars, up to 40 mm in length, that then pupate.

Voucher specimens of ants, moths (identified by Dr. M.R. Honey) and parasitoid wasps (identified by Dr. J. LaSalle) were deposited in the Museum of Natural History, London.

### The search for parasitized *Oecophylla longinoda* colonies

To evaluate the rate of parasitism by *E. albifascia* caterpillars, all of the nests of 205 *O. longinoda* colonies (N ≥ 20 to avoid incipient colonies) on small trees (<6 m tall) to permit easy access were first inspected externally to verify if clusters of *Eublemma* eggs had been laid on the leaves of the nests; if so, we counted the number of eggs. The nests were then opened to verify their content, including the presence of caterpillars (the last instars were counted), dealated queen(s), winged males and females as well as their pupae (plus large last instar female larvae). Although the ants were very alarmed, this process is not destructive (except for the colonies gathered for specific studies, see below) and the workers repaired the opened nests (see the opening of the nests and the observations inside artificial nests^32^). In addition, ≈ 40 more colonies were inspected during preliminary and complementary studies.

### Behavioral relationships between *Eublemma albifascia* caterpillars and *Oecophylla longinoda* workers

Laboratory studies were conducted on 13 *O. longinoda* colonies sheltering *Eublemma albifascia* caterpillars gathered in Yaoundé or Nkolbisson to avoid a high rate of caterpillar mortality from being transported over a long distance. Nests were collected by cutting down the supporting branches using a clipping pole, putting them into plastic bags and taking them to the laboratory. They were installed in 40 × 20 × 5 cm plastic boxes with a transparent cover (which we covered with a screen outside of observational periods to keep the workers from occulting this surface with silk) opening onto a table through holes 0.8 cm in diameter permitting the passage of emerging adult moths. The bases of the legs of the tables were placed in boxes filled with oil to prevent the workers from escaping. Extrafloral nectar-bearing potted plants (*Alchornea chordifolia* or *Hibiscus* spp.) were placed on the tables to permit the workers to forage for nectar and honeydew as they attend scale insects on these plants. The workers wove new nests in the foliage of these plants. The colonies were also provided *ad libitum* with water, honey and prey (mostly cricket larvae and small mealworms).

Although the workers became quickly used to being observed, most of the inside nest observations were conducted at night using red lighting. Other observations were conducted between 8:00 and 12:00 to record the number of cases when a major worker furnished trophic eggs to the queen in control colonies and to four last instar caterpillars in parasitized colonies.

To verify if *E. albifascia* caterpillars acquired the colony odor inside their host nests or, rather, have an intrinsic, appeasing odor for their host ants, we conducted two series of confrontation experiments based on the notion of colony mate recognition^11^. A control experiment was conducted using 40 *O. longinoda* workers transferred from one nest to another from the same colony and 40 others between two nests from two different colonies. We then transferred 20 caterpillars from one nest to another belonging to a neighboring, alien colony. Then, 20 other caterpillars were transferred from one nest to a distant one belonging to the same colony (two colonies used, one of them reared in artificial nests).

### The impact of caterpillar pressure on the sex ratio in *Oecophylla longinoda* colonies

All of the nests from each colony were gathered, put into large plastic bags and transported to the laboratory where they were placed in a refrigerator. Then, we counted the caterpillars in parasitized colonies as well as the pupae and winged males and females in both types of colonies. While doing so, we noticed that parasitized colonies can have a queen morphologically similar to those in control colonies (thereafter “normal queens”) or a queen with a “contracted gaster” (Fig. 1).

We first used a general linear model (GLM) (R v. 2.14.2 software; R Development Core Team used for all statistical comparisons) to test the hypothesis that highly parasitized colonies (N = 8; they sheltered 131, 139, 144, 151, 177, 184, 201 and 214 last instar caterpillars, respectively; Table 1) contained more males than did control colonies (N = 12; no caterpillar found whatever the instar; queens with a normal gaster). Because our response variable (i.e., the sex of the ants) was binary, we modeled it with a binomial error distribution and tested the effect of the presence of parasites on the probability of being a male. These two lots of colonies were composed of a similar number of nests (mean ± SE: 28.75 ± 2.00 ‘nests’ per control colony *vs*. 29.50 ± 2.65 ‘nests’ per parasitized colony; t = 0.23; 18 df; P = 0.82).

Second, in the four parasitized colonies having a queen with a contracted gaster (i.e., containing 206, 241, 260 and 274 last instar caterpillars, respectively; Table 1), the male pupae and adults were too numerous to be counted. So, we counted the pupae and winged females in the field and verified if worker pupae were present.

Third, the same process was applied to six parasitized colonies having lost their queen (i.e., containing 55, 64, 69, 288, 235 and 359 last instar caterpillars, respectively); some nests belonging to the first three colonies were full of agglutinated cocoons (Fig. 1 and Table 1). The numbers of pupae and winged males and females, which correspond to discrete, positive variables, were compared using a Poisson error distribution in a GLM.

After opening the nests of both the control colonies and the parasitized colonies, we noted the number of eggs surrounding the queen in each colony on an ordinal scale or gathered them using a fine paintbrush, and put them into a box to be counted in the laboratory. To test the effect of parasites on the number of eggs, we used the non-parametric Wilcoxon rank test to account for the ordinal nature of the response variable (Table 1).

### Starvation of *Oecophylla longinoda* queens in parasitized colonies

For two *O. longinoda* control colonies and four parasitized colonies bred in the laboratory, we conducted 54 series of 10-minute observations (9 h) during which we noted each time it occurred the duration of worker-queen trophallaxis in the former case and worker-caterpillar trophallaxis in the latter case (one caterpillar targeted in each colony during each observation session). The durations of the trophallaxis, an over-dispersed positive variable, were compared using a GLM with a quasi Poisson error distribution. The numbers of trophallactic events, a discrete and positive but not over-dispersed variable, were compared using a GLM with a Poisson error distribution.

For the same colonies, we also compared using a GLM with a Poisson error distribution the numbers of trophic eggs provided by major workers to the queens in control colonies and to caterpillars in parasitized colonies (20 series of 10-minute observations per queen and per caterpillar).

Using a microscale (Mettler® AE 260), we weighed minor, media and major workers, males, winged queens and mated queens (N = 10 in each case) taken from control colonies, and last instar caterpillars (N = 28) as well as “normal” queens (N = 5) and queens with a contracted gaster (N = 4) taken from parasitized colonies. The weight of 10 queens from control colonies and nine from parasitized colonies were compared with a classical GLM with a normal error distribution.

## Results

### The search for parasitized *Oecophylla longinoda* colonies

Of the 205 colonies monitored, 32 (15.61%) were parasitized by *E. albifascia* caterpillars, all containing 44 to 359 last instar individuals during the campaigns of investigation (Table 1; colonies parasitized by only earlier instars noted during preliminary studies).

The number of *E. albifascia* eggs per cluster varied between 86 and 133 (mean ± SE: 107.9 ± 3.5; N = 16; Fig. 1a). The highest number of last instar caterpillars per parasitized colony (335–359; Table 1) likely corresponds to three egg clusters laid concomitantly on different nests of the same colony (observed once). The lowest values (44 to 86 last instar caterpillars; Table 1), rather corresponded to clusters in which some eggs were parasitized by tiny parasitoid hymenopterans (Encyrtidae; *Ooencyrtus* sp.; emerging individuals were noted and captured once). Another parasitoid hymenoptera (*Pedobius* sp.; Elophidae) develops inside the caterpillars (individuals captured from laboratory-bred colonies).

### Behavioral relationships between *Eublemma albifascia* caterpillars and *Oecophylla longinoda* workers

**Figure 2:**
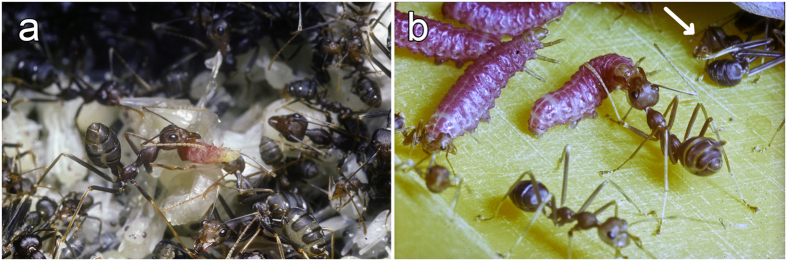
Behavioral relationships between *Eublemma albifascia* caterpillars and *Oecophylla longinoda* workers. (**a**) A second instar caterpillar riding beneath a worker’s head. (**b**) An intermediary instar caterpillar involved in a trophallactic exchange with a major worker while a major worker is in the process of laying a trophic egg to be provided to the caterpillar (see arrow).

We noted from laboratory breeding that the *O. longinoda* workers seized just-hatched first instar *E. albifascia* caterpillars with their mandibles and transported them to their colony, placing them among the brood (Fig. 1b). Larger, second instar caterpillars, their pink color contrasting with that of the brood and the workers (Fig. 2a), can remain among the brood. There, to obtain food, they use their abdominal prolegs to anchor themselves on an ant larva and bend their body upwards so that their head and thoracic legs reach the mouth parts of passing worker nurses that regurgitate food. Still using their abdominal prolegs, these caterpillars can anchor themselves under a worker’s head and steal food during inter-worker trophallaxis or, anchored on a worker’s head, thorax or gaster, move their thoracic legs to obtain trophallaxis from passing workers (Fig. 2a). Larger intermediary and last instar caterpillars, their abdominal prolegs anchored on the substrate, quasi permanently solicit food (Figs 1c and 2b). They obtain both regurgitations and trophic eggs from *Oecophylla* workers which generally circle around the anterior part of their body, taking turns in furnishing food (Fig. 2b). The workers frequently lick the body of each caterpillar and recuperate anal secretions (Fig. 1c).

All 20 last instar caterpillars transferred between nests belonging to the same colony were gently seized by their thoracic legs by one or two major workers which pulled them backward inside the new nests. We saw that those introduced into an artificial nest recovered their food-soliciting behavior after a few minutes. On the contrary, all 20 caterpillars transferred to a nest from an alien colony were fiercely attacked, and then spread-eagled and killed by several workers (a common behavior in *Oecophylla* during predation or territorial conflicts^1^,^25^). *Oecophylla longinoda* workers transferred between two nests belonging to the same colony were ignored, while all those transferred to an alien colony were attacked, spread-eagled and killed.

When emerging from their cocoons (observed 52 times in laboratory breeding), the adult moths, their wings still folded and crumpled, crawled outside the nest and looked for an isolated area, generally by climbing on one of the potted plants placed on the table bearing the artificial nests. There, they spread their wings (see an adult moth Fig. 1f). Most of the *O. longinoda* workers ignored them; occasionally a major attempted to bite them, but its mandibles closed on the long deciduous scales that cover them. These workers then spent a long time cleaning their mandibles while the newly eclosed adult moths remained unmolested.

### The impact of caterpillar pressure on the sex ratio in *Oecophylla longinoda* colonies

**Figure 3:**
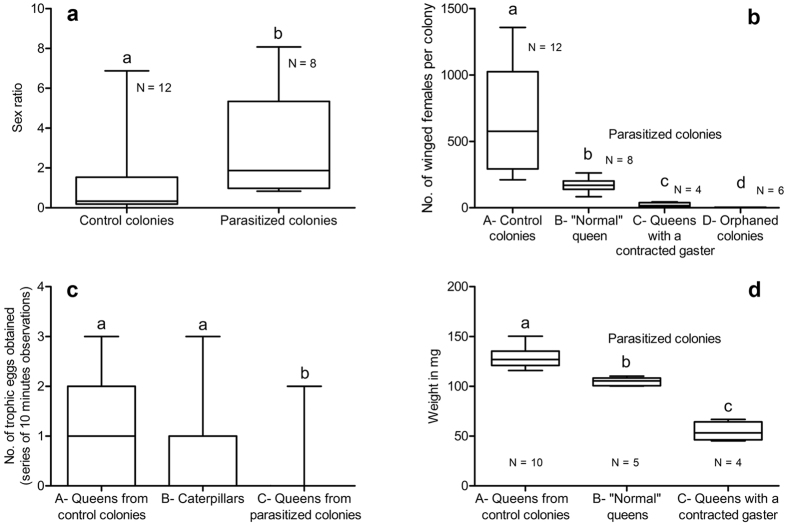
Comparisons concerning control, non-parasitized *Oecophylla longinoda* colonies and those parasitized by *Eublemma albifascia* caterpillars (Boxes represent the 25–75^th^ percentiles, the bar inside the boxes the medians, and the whiskers the minimum and the maximum values; different letters indicate significant differences at P < 0.001). (**a**) Differences in sex ratio between control and parasitized colonies (i.e., the queens had a contracted gaster) (GLM with Binomial error distribution: Z = 22.19; P < 0.001). (**b**) Comparison of the number of winged females (adults plus pupae) between control colonies and parasitized colonies with a “normal” queen (GLM with Poisson error distribution; Z = −45.74; P < 0.001), with queens having a contracted gaster (Z = −31.14; P < 0.001) and with orphaned colonies (Z = −8.27; P < 0.001) (orphaned colonies: colonies having lost their queen). (**c**) Comparison of the number of trophic eggs provided by major workers to the queens in two control colonies, the queens in four parasitized colonies (with a normally developed gaster) and the caterpillars in these parasitized colonies (N = 20 in each of these 10 situations; GLM with Poisson error distribution; queens from control colonies *vs*. caterpillars: Z = 1.68; NS; *vs*. queens from parasitized colonies: Z = −4.92, P < 0.001). (**d**) Comparison of the weights of queens between control colonies and parasitized colonies with a “normal” queen (GLM with normal error distribution; t = −4.66; P < 0.001) and with queens with a contracted gaster (t = −13.33; P < 0.001).

The number of last instar caterpillars represented a proxy of three levels of parasitism as the colonies can also shelter younger caterpillars. First, the queens in some parasitized colonies (i.e., 44 to 214 last instar caterpillars per colony; Table 1) seemed “normal” compared to those in control, non-parasitized colonies. Yet, colonies sheltering 131 to 214 caterpillars produced significantly more males than did the control ones, the sex ratio being male-biased (P < 0.001; Fig. 3a). Second, in parasitized colonies whose queen had a contracted gaster (i.e., 206 to 274 last instar caterpillars per colony; Table 1; Fig. 1g) the sex ratio was strongly male-biased (Fig. 3a). Third, parasitized colonies having lost their queen were split into two lots: sheltering as many as 288 to 359 last instar caterpillars, or as few as 55 to 69 but with numerous agglutinated cocoons (Table 1 and Fig. 1e). This time, female pupae and winged females were absent, while males abounded, their number likely surpassing that of the workers (Fig. 1d).

To these three levels of parasitism corresponded the obvious scarcity or even the absence of eggs surrounding the queens in parasitized colonies, while the control queens were surrounded by several egg piles (more than 100 eggs surrounding the queen in 12 cases out of 12 in the control colonies *versus* 3 cases out of 22 in parasitized colonies with a queen having a “normal” gaster; Wilcoxon rank test: P < 0.001; Table 1). Accordingly, we noted a significant decrease in the number of female pupae plus winged females at each step when comparing (1) control colonies to (2) parasitized colonies with a “normal” queen (P < 0.001), (3) to parasitized colonies whose queen had a contracted gaster (P < 0.001), and (3) to parasitized, orphaned colonies (P < 0.001; Fig. 3b).

### Starvation of *Oecophylla longinoda* queens in parasitized colonies

The workers, engaged in feeding the numerous caterpillars, completely neglected their queen. Moreover, each caterpillar benefited from significantly more trophallaxis than did the queens in control, non-parasitized colonies (161.1 ± 6.79 *vs*. 97.3 ± 16.4 trophallaxis for 9 h of observation; Z = −13.04: P < 0.001).Yet, the durations of worker-caterpillar trophallaxis were significantly lower than for worker-queen exchanges (1 to 268 seconds *vs*. 1 to 34 seconds; t = −17.29; P < 0.001). Concerning protein supply, the queens (with a “normal” gaster) in parasitized colonies were provided significantly fewer trophic eggs from major workers than were either the queens in control colonies or last instar caterpillars (P < 0.001), the difference between the two latter situations being non-significant (NS; Fig. 3c). Because the parasitized colonies contained 44 to 359 last instar caterpillars (not taking into account that younger caterpillars can be present) the queens of parasitized colonies were progressively starved. Accordingly, the queens in the control colonies were significantly heavier than the “normal” queens in the parasitized colonies (P < 0.001) and the queens with a contracted gaster (P < 0.001; Fig. 3d).

Because ants and moths are holometabolous and the diet of the *E. albifascia* caterpillars presents strong similarities with that of their host *O. longinoda*, particularly the queens, we estimated the trade-off between the production of caterpillars and that of workers, males and winged females (Table 2). For example, feeding 44 caterpillars (the lowest recorded value) corresponds to the production of 1,614.8 winged females or more than the maximum number of female pupae plus winged females noted in control colonies in a snapshot control (i.e., 1,358 individuals; Table 1); the highest number, 359 caterpillars, corresponds to the production of 13,175.1 winged females or 9.7 times the above-cited maximum number.

## Discussion

That *E. albifascia* caterpillars obtain food through a cuckoo-like behavior is, to the best of our knowledge, a new finding in moths and therefore represents an evolutionary convergence with lycaenid butterflies. Furthermore, second instar caterpillars, able to ride on the workers’ bodies, can steal food while their transporting worker is engaged in trophallaxis with a nestmate or are able to solicit passing workers and thus possess a set of behaviors reminiscent of that of myrmecophilous gamasid mites of the genus *Antennophorus*^33^. The intermediary-to-last instar caterpillars are not only provided energy-rich food by the workers lining up to feed them, they also obtain trophic eggs, something reported for lycaenids with a cuckoo-like parasitic behavior^4^,^34^.

*Eublemma* females short-circuit the adoption process by laying eggs directly on the ant nests, and just-hatched caterpillars are carried by ant workers inside their nest. The caterpillars of only a few Theclinae and Miletinae lycaenids are obligate parasites of ant colonies from their first instar onwards (i.e., without a phytophagous or a coccidophagous first phase^35^,^36^).

As shown for several intranidal myrmecophilous parasites, *E. albifascia* caterpillars likely acquire the specific colony odor inside their host ant nests; such mimicry results in their treatment as colony members^3^,^4^,^13^,^18^.

The cuckoo-like strategy leads the host *O. longinoda* colonies to ruin as the queen is neglected to the benefit of the *E. albifascia* caterpillars. Indeed, each of them seems to take the queen’s place, obtaining more trophic eggs and more trophallaxis (but for a shorter duration) than the queen in a control colony. Furthermore, this effect is multiplied by their number (44 to 359 caterpillars per colony). Consequently, *E. albifascia* caterpillars exert so strong a pressure on their host colonies that the process generally ends in the death of the queen and progressively of the colony. This is in contrast to the cuckoo-like myrmecophily of lycaenid caterpillars which has less deleterious consequences for their host colony, especially compared to those which prey on their hosts’ brood^37^. Also, *P. alcon* and *P. arion* caterpillars can imitate the acoustical signals of their host *Myrmica* queens, obtaining in this way a high status in the host ant colony hierarchy^14^,^15^.

As for myrmecophilous lycaenids^4^, *E. albifascia* caterpillars are regulated by parasitoid hymenopterans. Furthermore, *E. albifascia* is attacked by both egg and larval parasitoids, which may have had important consequences on *Oecophylla* abundance at our study sites^38^.

Because *O. longinoda* queens are starved in colonies parasitized by *E. albifascia* caterpillars, their weight decreased and their gaster become contracted while the number of last instar caterpillars increase. So, the queens lay fewer and fewer eggs that are the vehicle for the pheromones that induce infertility in workers. Indeed, these inhibiting pheromones are composed of hydrocarbons that cover the eggs which are distributed to the different nests of the colony^39^,^40^,^41^,^42^. Consequently, workers lay male-destined haploid eggs rather than laying trophic eggs. Accordingly, we noted a decrease in the number of winged females (pupae and adults) accompanied by an increased production of males, likely from workers, and, so, a change in the sex ratio. After the death of the queen, the workers continued to lay eggs and to produce males (see also Hölldobler & Wilson^1^), something that allowed the colony to invest its remaining resources before it vanished entirely.

In conclusion, the caterpillars of most *Eublemma* species (Noctuidae; Acontiinae) are specialized coccid predators^3^,^19^,^20^, the latter frequently found associated with ants, so that this ability is likely one of the prerequisites in the evolution toward myrmecophily^21^,^22^. Indeed, a convergence exists with the subfamily Melitinae (Lycaenidae) whose caterpillars are all aphytophagous; certain of them, specialized Hemiptera feeders, have derived semiochemicals that lure ants, while others became parasites of ant colonies^35^.

## Additional Information

**How to cite this article**: Dejean, A. *et al.* A cuckoo-like parasitic moth leads African weaver ant colonies to their ruin. *Sci. Rep.* **6**, 23778; doi: 10.1038/srep23778 (2016).

## Figures and Tables

**Table 1:** Composition of the colonies studied.

Control, non-parasitized colonies (all have a queen)							Colonies parasitized by *Eublemma albifascia* pupae								
No. of queens	No. of ♂	No. of ♀	Σ♂/Σ♀(n + w♀)	Sex ratio	Worker pupae	No. eggs*	No. of caterpillars per colony	No. of queens	No. of ♂	No. of ♀	Σ♂/Σ♀	Sex ratio	Worker pupae	No. eggs*	
1	0	672	0/672	0	Yes	>100	69	0	TNTC	0	TNTC/0	∞	0	/	Orphaned coloniesNumerous moth cocoons
1	1450	211	1450/211	6.87	Yes	>100	64	0	TNTC	0	TNTC/0	∞	0	/	
1	766	428	766/428	1.79	Yes	>100	55	0	TNTC	0	TNTC/0	∞	0	/	
1	199	1358	199/1358	0.15	Yes	>100	359	0	TNTC	0	TNTC/0	∞	0	/	Fewer or absence of moth cocoons
1	344	1081	344/1081	0.32	Yes	>100	335	0	TNTC	0	TNTC/0	∞	A few	/	
1	234	741	234/741	0.31	Yes	>100	288	0	TNTC	0	TNTC /0	∞	A few	/	
1	865	1130	865/1130	0.76	Yes	>100	274	1*	TNTC	10	TNTC/10	M-b	A few	0	* Queen with a contracted gaster
1	42	853	42/853	0.05	Yes	>100	260	1*	TNTC	44	TNTC/44	M-b	A few	0	
1	80	273	80/273	0.29	Yes	>100	241	1*	TNTC	22	TNTC/22	M-b	A few	0	
1	1137	3518	1137/351	3.24	Yes	>100	206	1*	TNTC	6	TNTC /6	M-b	A few	0	
1	78	232	78/232	0.33	Yes	>100	214	1	793	140	793/140	5.66	Yes	22	Queen present, its gaster seems “normal”
1	305	479	305/479	0.64	Yes	>100	201	1	604	138	604/138	4.37	Yes	0	
							184	1	291	207	291/207	1.41	Yes	23	
							177	1	415	178	415/178	2.33	Yes	≈ 30	
							151	1	303	262	303/262	1.16	Yes	≈ 50	
							144	1	172	187	172/187	0.919	Yes	≈ 60	
							139	1	670	83	670/83	8.08	Yes	≈ 80	
							131	1	133	160	133/160	0.831	Yes	≈ 80	
							249	1	Yes	Yes	Yes	/	A few	24	Only presence - absence notedQueen present, its gaster seems “normal”
							230	1	Yes	Yes	Yes	/	Yes	0	
							191	1	Yes	Yes	Yes	/	Yes	28	
							169	1	Yes	Yes	Yes	/	Yes	≈ 80	
							163	1	Yes	Yes	Yes	/	Yes	≈ 50	
							155	1	Yes	Yes	Yes	/	Yes	≈ 80	
							128	1	Yes	Yes	Yes	/	Yes	≈ 50	
							121	1	Yes	Yes	Yes	/	Yes	17	
							112	1	Yes	Yes	Yes	/	Yes	≈ 30	
							98	1	Yes	Yes	Yes	/	Yes	≈ 50	
							86	1	Yes	Yes	Yes	/	Yes	>100	
							74	1	Yes	Yes	Yes	/	Yes	≈ 60	
							49	1	Yes	Yes	Yes	/	Yes	>100	
							44	1	Yes	Yes	Yes	/	Yes	>100	

TNTC: too numerous to count under field monitoring; M-b: male-biased situation; No. eggs*: number of eggs in the queen’s chamber.

All parasitized colonies contained last instar caterpillars, but some with only second and/or intermediary instar caterpillars were recorded during the preliminary and additional studies.

**Table 2:** Mean weight (±SE) in mg of last instar *Eublemma albifascia* caterpillars and the different castes and sub-castes produced by the host *Oecophylla longinoda* colonies and proxy of the equivalent number of individuals from these castes and sub-castes corresponding to the feeding of one caterpillar prior to its pupation as well as 44, 159 and 359 caterpillars (the minimum, the median and the maximum values noted for parasitized colonies).

Last instar caterpillars	Winged females	Major workers	Media workers	Minor workers	Males
3725.0 ± 224.7	101.5 ± 3.8	12.6 ± 0.38	7.9 ± 0.46	2.4 ± 0.09	7.1 ± 0.25
	Proxy of ratios				
One last instar caterpillar ≈	37	296	472	1552	525
x 44 ≈	1,615	13,008	20,747	68,292	23,085
x 159 ≈	5,835	47,006	74,971	256,781	83,419
x 359 ≈	13,175	106,133	169,275	557,198	188,349
